# Protective Antimicrobial Effect of the Potential Vaccine Created on the Basis of the Structure of the IgA1 Protease from *Neisseria meningitidis*

**DOI:** 10.3390/vaccines12121355

**Published:** 2024-11-30

**Authors:** Yuri Prokopenko, Alexei Zinchenko, David Karlinsky, Olga Kotelnikova, Olga Razgulyaeva, Elena Gordeeva, Elena Nokel, Oxana Serova, Elena Kaliberda, Larisa Zhigis, Lev Rumsh, Ivan Smirnov

**Affiliations:** 1 Laboratory of Antibiotic Resistance, Shemyakin-Ovchinnikov Institute of Bioorganic Chemistry of the Russian Academy of Sciences, 117997 Moscow, Russia; tetrahydrofuran@mail.ru; 2 Laboratory of Proteolytic Enzyme Chemistry, Shemyakin-Ovchinnikov Institute of Bioorganic Chemistry of the Russian Academy of Sciences, 117997 Moscow, Russia; alezina@mail.ru (A.Z.); ovkot.2003@mail.ru (O.K.); lgor@mail.ru (E.G.); nea.ibch@gmail.com (E.N.); oxana.serova@gmail.com (O.S.); elena.kaliberda@ibch.ru (E.K.); lrumsh@mail.ru (L.R.); ivansmr@inbox.ru (I.S.); 3 Laboratory “Polymers for Biology”, Shemyakin-Ovchinnikov Institute of Bioorganic Chemistry of the Russian Academy of Sciences, 117997 Moscow, Russia; raz-olga-a@yandex.ru (O.R.); larisa.zhigis@gmail.com (L.Z.); 4 Laboratory of Protein Chemistry, Lomonosov Moscow State University, 119991 Moscow, Russia; 5 Laboratory of Biotechnology of Recombinant Hormonal Drugs, Endocrinology Research Centre, 117292 Moscow, Russia

**Keywords:** *Neisseria meningitidis*, IgA1 protease, epitopes, protein structure prediction, fused proteins, meningococcal vaccine

## Abstract

**Background/Objectives:** IgA1 protease is one of the virulence factors of *Neisseria meningitidis*, *Haemophilus influenzae* and other pathogens causing bacterial meningitis. The aim of this research is to create recombinant proteins based on fragments of the mature IgA1 protease A^28^–P^1004^ from *N. meningitidis* serogroup B strain H44/76. These proteins are potential components of an antimeningococcal vaccine for protection against infections caused by pathogenic strains of *N. meningitidis* and other bacteria producing serine-type IgA1 proteases. **Methods:** To obtain promising antigens for creating a vaccine, we designed and obtained several recombinant proteins. These proteins consisted of single or directly connected fragments selected from various regions of the IgA1 protease A^28^–P^1004^. The choice of these fragments was based on our calculated data on the distribution of linear and conformational B-cell epitopes and MHC-II T-cell epitopes in the structure of IgA1 protease, taking into account the physicochemical properties of potential compounds and the results of a comparative analysis of the spatial structures of the original IgA1 protease and potential recombinant proteins. We studied the immunogenic and protective effects of the obtained proteins on the BALB/c mice against meningococci of serogroups A, B and C. **Results:** Proteins MA^28^–P^1004^-LEH_6_, MW^140^–K^833^-LEH_6_, MW^329^–P^1004^-LEH_6_, M(W^140^–H^328^)-(W^412^–D^604^)-(Y^866^–P^1004^)-LEH_6_ and M(W^140^–Q^299^)-(Y^866^–P^1004^)-LEH_6_ have shown the following antibody titers, 10^3^/titer: 11 ± 1, 6 ± 2, 6 ± 1, 9 ± 1 and 22 ± 3, respectively. Also, the last two proteins have shown the best average degree of protection from *N. meningitidis* serogroups A, B and C, %: 62 ± 6, 63 ± 5, 67 ± 4 respectively for M(W^140^–H^328^)-(W^412^–D^604^)-(Y^866^–P^1004^)-LEH_6_ and 70 ± 5, 66 ± 6, 83 ± 3 respectively for M(W^140^–Q^299^)-(Y^866^–P^1004^)-LEH_6_. **Conclusions:** We selected two recombinant proteins consisting of two (M(W^140^–Q^299^)-(Y^866^–P^1004^)-LEH_6_) or three (M(W^140^–H^328^)-(W^412^–D^604^)-(Y^866^–P^1004^)-LEH_6_) linked fragments of IgA1 protease A^28^–P^1004^ as candidate active component for an antimeningococcal vaccine.

## 1. Introduction

Bacterial meningitis is a severe neuroinfectious disease caused by various pathogens. It is difficult to diagnose in the early stages, making timely treatment difficult and causing severe complications with a high mortality rate. Therefore, vaccination is the most effective method of combating the pathogens of this disease [1,2].

The main causative agents of bacterial meningitis are the pathogenic bacteria *Neisseria meningitidis*, *Haemophilus influenzae*, *Streptococcus pneumoniae* and *Streptococcus agalactiae* (group B). *N. meningitidis* causes the most common and dangerous form of bacterial meningitis. Based on the structure of capsular polysaccharide antigens, this pathogen is classified into 13 serogroups, six of which (A, B, C, W, X, Y) are epidemically dangerous [3].

Currently, three types of vaccines are used in medical practice: (1) polysaccharide vaccines—bivalent (against *N. meningitidis* serogroups A and C), trivalent (against A, C and W) and quadrivalent (A, C, W and Y); (2) conjugate vaccines—monovalent A, monovalent C, quadrivalent (A, C, W and Y) and pentavalent (A, C, W, Y, X); (3) vaccines based on pathogen surface proteins—against *N. meningitidis* serogroup B [1,4,5,6]. All of these vaccines are either multicomponent or directed against one specific pathogen. According to WHO, there is currently no universal vaccine against bacterial meningitis of various etiologies [https://www.who.int/teams/immunization-vaccines-and-biologicals/diseases/meningitis (accessed on 1 August 2024)].

One of the possible protective antigens, the structure of which can be used to design compounds that can become the basis of a universal broad-spectrum vaccine, is the bacterial IgA1 protease, an important virulence factor of a number of pathogens [7]. The prospects for using this enzyme and its fragments as an active ingredient of a universal monovaccine are based on the high similarity of the primary structures of serine-type IgA1 proteases from *N. meningitidis* of various serogroups, *N. gonorrhoeae* and a number of strains of *H. influenzae* [8,9].

These highly specific enzymes catalyze the hydrolysis of Pro-Ser or Pro-Thr peptide bonds in the hinge region of secretory (sIgA1) and serum (IgA1) human immunoglobulins A1 [10]. There is also data on the cleavage of IgG3 by type 1 IgA1 protease from *N. meningitidis* [11]. By cleaving sIgA1, which covers the mucous membrane of the host organism, IgA1 proteases facilitate the adhesion of pathogenic bacteria to the surface of the mucous membrane, and thus promote the development of infection. Immunization of the host organism with recombinant IgA1 protease should lead to the formation of antibodies, which neutralize the IgA1 protease of microorganisms and prevent the development of infection at its early stage, and to the subsequent formation of long-term immunity.

We have previously shown that immunization of animals with native IgA1 protease from meningococcus serogroup A [12], active or mutant at the serine-267 residue (Ser267Ala) recombinant IgA1 protease from meningococcus serogroup B [13,14], and some recombinant proteins based on fragments of IgA1 protease A^28^–P^1004^ from *N. meningitidis* serogroup B strain H44/76 [15,16,17,18], effectively protected animals from infection with a live virulent culture of meningococci of serogroups A, B and C, and protective antibodies, formed during immunization of animals with recombinant IgA1 protease, are able to bind not only to the secreted enzyme, but also to IgA1 protease exposed on the surface of microbial cells [15]. In addition, it is known from the literature that class G immunoglobulins, isolated from the serum of patients infected with pneumococcus, neutralized the activity of streptococcal metallo-type IgA1 protease on the surface of bacterium *S. pneumoniae* [19], and immunization of mice with recombinant IgA1 protease from *Streptococcus suis* serotype 2 led to the formation of specific antibodies and provided complete protection of animals from infection with a lethal dose of this streptococcus [20].

These data allowed us to consider the recombinant IgA1 protease MA^28^–P^1004^-LEH_6_ and recombinant proteins, based on fragments of its primary structure and possessing immunogenic and protective activity, as promising candidate molecules for creating a broad-spectrum vaccine. Such a vaccine is presumed to be able to provide effective protection against infectious diseases caused by the vast majority of pathogenic strains of *N. meningitidis* of various serogroups, as well as some other microorganisms producing serine-type IgA1 proteases.

The aim of this research is to study the role of various parts of the structure of the *N. meningitidis* IgA1 protease in inducing the immunogenic and protective properties of this enzyme, and to create new recombinant proteins—potential components of a broad-spectrum vaccine for long-term effective protection against infectious diseases caused by pathogenic strains of *N. meningitidis* and other bacteria producing serine-type IgA1 proteases.

## 2. Materials and Methods

### 2.1. Calculations

#### 2.1.1. Data on the Primary Structure of IgA1 Protease

This work uses a description of the primary structure of the full-length IgA1 protease from *Neisseria meningitidis* serogroup B strain H44/76, presented on the website of the National Center for Biotechnology Information [21] in the GenBank database [22] under code ADC80147.1 (https://www.ncbi.nlm.nih.gov/protein/289063650/ (accessed on 25 October 2016)). This autotransporter’s polypeptide fragment with the primary structure A^28^–P^1004^, containing the protease subdomain and the gamma peptide, was used as the basic amino acid sequence in calculations and further studies.

#### 2.1.2. Predicting the Location of T-Cell Epitopes

Calculations to predict the location of MHC-II T-cell epitopes in the primary structure of the A^28^–P^1004^ fragment of IgA1 protease ADC80147.1 for human and BALB/c mouse hosts were carried out at IEDB Analysis Resource [23] (https://iedb.org/ (accessed on 22 February 2023)) using program “T Cell Epitopes—MHC Binding Prediction/Peptide binding to MHC class II molecules” with method “IEDB recommended 2.22” [24,25,26,27,28,29]. The calculation parameters include the peptide length of 15 aa, the H2-IAd and H2-IEd alleles for mice and the “Full HLA reference set” [30] for humans. For the human hosts, the lists of “epitope-allele” pairs in the “rank ≤ 10” and “rank ≤ 20” variants, were formed into lists corresponding to the amino acid sequence A^28^–P^1004^ sections to be considered, and used as input data for Population Coverage [31] program presented on the IEDB website:

- Via an online server (accessed on 3 April 2023) for the amino acid sequences of IgA1 protease A^28^–P^1004^, its fragments: E^135^–H^328^, W^329^–H^622^, H^835^–P^1004^, W^140^–K^833^, W^329^–P^1004^ and potential fusion structures (W^140^–H^328^)-(W^412^–D^604^)-(Y^866^–P^1004^), (W^140^–Q^299^)-(Y^866^–P^1004^);
- Using the downloadable version (version 3.0.2) of the program—separately for each epitope located in the A^28^–P^1004^ chain.

The world as a whole, Russia, and the following regions were selected as the geographical regions under consideration: Europe, West Indies, East Asia, Northeast Asia, South Asia, Southeast Asia, Southwest Asia, East Africa, West Africa, Central Africa, North Africa, North America, Central America, South America, and Oceania. The calculation results in the Population Coverage program exclude the alleles HLA-DRB3*01:01, HLA-DRB3*02:02, HLA-DRB4*01:01 and HLA-DRB5*01:01.

#### 2.1.3. Modeling Three-Dimensional Structures

For the IgA1 protease from *N. meningitidis* serogroup B strain H44/76 (GenBank: ADC80147.1) in the full-length form, in the mature form A^28^–P^1004^ and in the recombinant form MA^28^–P^1004^-LEH_6_ (protein 1), as well as for the recombinant proteins, designed on the basis of fragments of the primary structure of this enzyme, with formulas: ME^135^–H^328^-LEH_6_, MW^329^–H^622^-LEH_6_, MH^835^–P^1004^-LEH_6_, MW^140^–K^833^-LEH_6_ (protein 2), MW^329^–P^1004^-LEH_6_ (protein 3), M(W^140^–H^328^)-(W^412^–D^604^)-(Y^866^–P^1004^)-LEH_6_ (protein 4) and M(W^140^–Q^299^)-(Y^866^–P^1004^)-LEH_6_ (protein 5), models of three-dimensional structures were built in AlphaFold2 [32] program, using ColabFold v1.5.2 [33] (no template information is used; for all proteins except the full-length one, the top ranked structure is relaxed using AMBER). Analysis of the quality of the resulting models was carried out using AlphaFold2 built-in tools (predicted Local Distance Difference Test, pLDDT). The Swiss PDB Viewer 4.1.0 [34] program was used to fit and compare (by RMSD between Cα atoms) sections of the models.

#### 2.1.4. Prediction of the Location of B-Cell Epitopes

The location of B-cell epitopes was predicted based on data on the tertiary structure of the A^28^–P^1004^ fragment of the model of the full-length IgA1 protease ADC80147.1, constructed using the AlphaFold2 method as described in Section 2.1.3, for linear and conformational B-cell epitopes using the ElliPro [35] method (on the IEDB online server), and for conformational B-epitopes using methods DiscoTope 2.0 [36] (on online servers IEDB and DTU Health Tech, https://www.healthtech.dtu.dk/) and SEPPA 2.0 [37] (secreted antigen, Homo and Mus hosts, on the online server of the Cao Zhiwei laboratory (http://www.badd-cao.net/seppa2/index.php) (all servers used in Section 2.1.4. were accessed on 13 March 2023). Parameters not listed here were set by default.

### 2.2. Experiment

#### 2.2.1. Production of Recombinant Proteins

A recombinant IgA1 protease with the general formula MA^28^–P^1004^-LEH_6_ and recombinant proteins based on fragments of the primary structure of this enzyme: ME^135^–H^328^-LEH_6_, MW^329^–H^622^-LEH_6_, and MH^835^–P^1004^-LEH_6_ were obtained as described in the article [17]. Fusion recombinant proteins 4 and 5, containing three and two fragments from different regions of the primary structure of the molecule MA^28^–P^1004^-LEH_6_, respectively, were obtained as described in the patent RU 2701964 C2 [38]. Recombinant proteins 2 and 3 were produced as described below.

To clone the DNA sequence encoding the IgA1 protease fragment W^140^–K^833^ (recombinant protein 2) into the pET-28(b+) vector, the *iga* gene was amplified according to the standard PCR protocol, using pET-31-IgA1-1004 DNA as a template. Using oligonucleotides IgA1-W140—5′-TGGCATCACGGAAATCAAGGTC-3′ and IgA1-K833-Xho—5′-GCGCTCGAGTTTGCTATGTTGGTTTAGGCTGA-3′ (forward and reverse primers), a DNA fragment of about 2100 bp in length was obtained. The PCR target product was treated with *Xho*I restriction enzyme and cloned into the pET-28b(+) vector (Novagen, Darmstadt, Germany) using *Nco*I (blunted) and *Xho*I unique sites of restriction so that the produced protein contained a His-tag in the C-terminal region.

Cloning of the IgA1 protease fragment W^329^-P^1004^ (recombinant protein MW^329^–P^1004^-LEH_6_) into the pET-28(b+) vector was carried out as described above, using oligonucleotides IgA1-W329—5′-TGGAACATTACTTTTGGCACAAA-3′ and IgA1-Xho-1004—5′-GCGCTCGAGAGGCGGTGCGACGACGATAT-3′.

Treatment of the PCR fragment and vector with restriction enzymes, ligation and transformation of competent cells were carried out using standard procedures. Sequencing of the isolated plasmid DNA confirmed the identity of the cloned sequences to the target sequence of the *iga* gene.

*E. coli* BL21(DE3)/*iga* strains were obtained by introducing pET-28(b+)–W^140^–K^833^/*iga* and pET-28(b+)–W^329^–P^1004^/*iga* vectors of plasmid DNA into competent cells of *E. coli* BL21(DE3) by heat shock method.

To obtain the producer strains pET-28(b+)–W^140^–K^833^/iga and pET-28(b+)–W^329^–P^1004^/iga (each species separately), 1 L of liquid LB medium containing 50 μg/mL kanamycin was added 1% of the corresponding overnight cell culture inoculum and grown at 37 °C on a shaker at 200 rpm for 2 h until reaching an optical density of 0.8 at a wavelength of 600 nm. The inducer isopropyl β-D-1-thiogalactopyranoside (IPTG) was then added to a final concentration of 0.5 mM, and incubation was continued under the same conditions for two hours. To collect cells, 1 L of culture medium of each type of producer strain was centrifuged at 3500 rpm. To isolate recombinant proteins 2 and 3, cell pellets (from 1 L of induced culture) were resuspended in a sevenfold volume of 20 mM Tris-HCl buffer solution with 0.15 M NaCl, pH 8.5. The cell suspension was treated with ultrasound five times for 1 h and centrifuged (20,000 × *g*). The insoluble part of the cell lysate was separated. The sediment and supernatant were analyzed for the content of target proteins by electrophoresis in 10% PAGE. It was shown that the target proteins are located in inclusion bodies.

#### 2.2.2. Purification of Recombinant Proteins

Inclusion bodies were isolated and the desired proteins were purified using the modified method [17]. Washed inclusion bodies were dissolved in 8 M urea and fractionated by chromatography on Ni-agarose (HiTrap^TM^ Chelating HP, Cytiva, Marlborough, MA, USA).

Protein, eluted from Ni-agarose under conditions of an increasing gradient of imidazole in 8 M urea, was subjected to stepwise dialysis against buffer solutions with decreasing urea concentrations. Renatured protein was purified sequentially by Q-Sepharose chromatography (HiTrap^TM^ Q FF, Cytiva, Marlborough, MA, USA) and reversed-phase HPLC (Delta-Pak^TM^ HPI C4 column, 300 Å, 5 μm, Waters, Milford, MA, USA) under conditions of increasing gradient of acetonitrile in 0.1% trifluoroacetic acid solution. Chromatographically pure recombinant proteins were dried in a freeze dryer. The purified proteins were stored at −20 °C.

The homogeneity and approximate molecular mass of the resulting preparations were confirmed by SDS-PAGE (10%) under denaturing conditions [39].

Molecular masses, theoretical pI and other physicochemical properties of the recombinant proteins were calculated using the server https://www.protparam.net/index.html (accessed on 5 June 2020) [40]. Amino acid sequences of the recombinant proteins were determined on the basis of nucleotide sequence of the cloned DNA.

The concentration of purified proteins in working solutions was determined by UV absorbance at 280 nm using the extinction coefficient calculated for each protein using the server https://www.protparam.net/index.html (accessed on 5 June 2020) [40].

#### 2.2.3. Assessment of Immunogenic and Protective Activity of Truncated Recombinant Proteins

The immunogenic potency and protective effect of the recombinant proteins were assessed on female BALB/c mice (16–18 g), aged 6–7 weeks, obtained from the Bioresource Collection of the Branch IBCh RAS (Pushchino, Moscow Region) supported by the Ministry of Science and Higher Education of the Russian Federation (Contract # 075-15-2021-1067). Mice were kept according to IACUC protocol (“IBCH 58a”, 15 May 2015).

Mice were immunized intravenously into the retro-orbital sinus twice at a dose of 0.002 g/kg of the studied protein at a concentration of 0.2 mg/mL with an interval of 45 days without the use of adjuvants or other fillers. At different times after immunization, 100 μL of blood was collected from the retro-orbital sinus using a capillary. The experimental group size was 10 animals for each protein.

In all experiments, the results obtained for the recombinant proteins were compared with the results for MA^28^–P^1004^-LEH_6_ as a reference antigen.

The presence of specific antibodies to MA^28^–P^1004^-LEH_6_ was assessed by solid-phase enzyme-linked immunosorbent assay (ELISA). 0.1 mL of MA^28^–P^1004^-LEH_6_ in Na-carbonate buffer (20 mM Na_2_CO_3_, 20 mM NaHCO_3_, pH 9.6) at a concentration of 20 μg/mL were bound to the wells of a 96-well Nunc Maxisorp plate (Thermofisher, Waltham, MA, USA) and incubated for 16 h at 4 °C. Next, 150 μL of blocking solution (0.1% casein in PBS with 0.1% Tween-20 (PBST 0.1%), pH = 7.4) were added to the antigen-coated wells and incubated for 2 h at 37 °C. The wells were washed four times with phosphate-buffered saline containing 0.1% Tween-20 PBST. Then, 0.1 mL of serum samples in double dilutions in blocking buffer solution, starting with a 1:10 dilution, were added to the wells. After incubation of the plate for 1 h at 37 °C, the serum solutions were removed, washed four times with PBST solution, and 0.1 mL of a solution of conjugate horseradish peroxidase—goat antibodies to total mouse immunoglobulins IgA or IgG were added to each well. 0.1 mL of a solution (50% orthophenylenediamine in citrate buffer solution, pH 5, contained 0.014% hydrogen peroxide) was added to each well and incubated for 10 min. The reaction was stopped by adding 50 μL of 0.3 M H_2_SO_4_. Optical density was determined at a wave length of 492 nm using Thermo Scientific Varioskan^®^ Flash (Thermo Fisher Scientific, Waltham, MA, USA) microplate reader for ELISA.

Antibody titers were calculated from the optical densities measured for the sera of immunized mice in comparison with the corresponding values obtained from titration of sera from non-immunized animals.

To infect animals, we used live virulent cultures of meningococcus of serogroups A (strain A208), B (strain H44/76) or C (strain C0638) at a dose of 0.25 × 10^6^ microbial cells (m.c.). The level of bacteremia in infected mice was assessed 4 h after infection [14].

The microbial culture was grown on a dense Tryp Case Soy Agar (TSA-F) nutrient medium with 5% calf blood serum at +37 °C, in a 5–10% CO_2_ atmosphere and high humidity (98%). After incubation, the colonies were washed off the surface of the nutrient medium with an isotonic NaCl solution and a suspension was prepared with a concentration of 1109 m.c./mL, which corresponds to an optical density of 0.23 at a wavelength of 610 nm on a spectrophotometer Eppendorf BioSpectrometer Kinetic (Eppendorf, Hamburg, Germany). Then, the resulting suspension of microbes was diluted with a solution of iron dextran (Sigma, Livonia, MI, USA), pH 7.2, at a temperature of +37 °C to a concentration of 0.5 × 10^6^ m.c./mL based on 1.6 mg/mL of iron.

Mice were infected with a microbe suspension of intraperitoneally in a volume of 0.5 mL. Four hours after infection, 10 μL of blood was individually collected from the retroorbital sinus of mice using a capillary. By serial dilutions in round-bottomed 96-well plastic plates, the obtained blood was diluted with an isotonic NaCl solution by 1125 times and 50 μL of the resulting solution was seeded in 12-well plates with a solid nutrient medium TSA-F with 5% calf serum. The plates with the microbial inoculation were incubated at +37 °C in an atmosphere of 5% CO_2_ for 18 h. After the end of incubation, the number of colony-forming cells (CFU) was counted for each blood sample and the average number of CFU in the group was calculated.

The activity of the proteins administered to mice was assessed by the level of bacteremia (the number of CFU in the blood) in immunized animals after infection compared with these indicators in the control group (non-immunized mice).

The protective activity of the proteins was assessed by the level of bacteremia in immunized mice in comparison with control (not immunized) mice using the following formula: 100% − CFU^experimental^/CFU^control^ × 100%.

Statistical processing of the results was carried out in OriginPro 2021 (OriginLab Corporation, USA), using ANOVA statistics with Bonferroni test (One-Way ANOVA for antibody titer analysis and Two-Way ANOVA for protectivity analysis).

## 3. Results

### 3.1. Distribution of T-Cell Epitopes in the Primary Structure of IgA1 Protease

In calculations predicting the locations of MHC-II T-cell epitopes, with length of 15 aa, in the primary structure of IgA1 protease A^28^–P^1004^ for human and mouse hosts (see Section 2.1.2), as a criterion for selecting an epitope–allele combination, we used the percentile_rank parameter (proposed in the “IEDB recommended 2.22” method of the “T Cell Epitopes—MHC Binding Prediction/Peptide binding to MHC class II molecules” program), further referred to as “rank”. When sorting epitope–allele pairs by rank, from lowest to highest, the top 10% of pairs have a rank less than or equal to approximately 20 (on the border of the top 10%, the rank is about 19–21).

**Figure 1:**
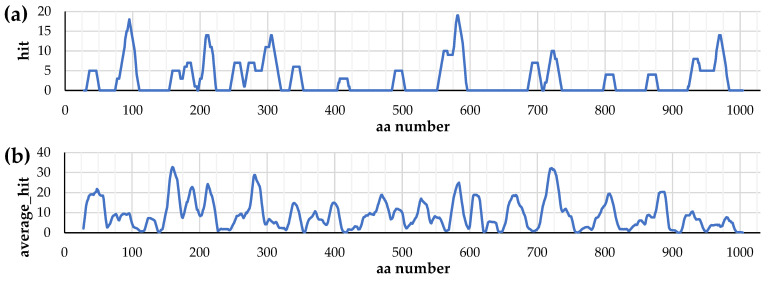
(**a**) The number of hits for amino acid residues involved in the predicted combinations of MHC-II T-cell epitopes, located on the A^28^–P^1004^ chain, with alleles of BALB/c mouse; rank ≤ 20. (**b**) The average_hit value sums for amino acid residues involved in the predicted combinations of MHC-II T-cell epitopes, located on the A^28^–P^1004^ chain, with alleles of human worldwide populations; rank ≤ 20.

The results for the predicted best-ranked (rank ≤ 20) pairs of MHC-II T-cell epitopes with mouse BALB/c alleles are shown in Figure 1a and Figure S1. They can be used to evaluate the effectiveness of a particular section of the amino acid sequence of the IgA1 protease A^28^–P^1004^ in inducing a specific immune response in mice of this strain. The obtained data analysis, taking into account the “rank ≤ 20” epitope–allele pair density in the selected regions, allows to suggest that the enzyme sequence fragments most promising from this point of view are 75–109, 155–352, 552–595, 686–735 and 923–983.

To assess the possible effectiveness of IgA1 protease A^28^–P^1004^ and its sections in the active ingredient of a vaccine intended for different human populations, we used the Population Coverage program. We chose average_hit (average number of epitope hits/HLA combinations recognized by the population) as the main parameter to estimate this effectiveness. For each residue on the A^28^–P^1004^ chain, we calculated a sum of average_hit values of the epitopes in which that residue is involved; the resulting diagrams for human populations from various geographical regions are shown in Figure S2a for combinations with rank ≤ 20 and in Figure S2b for those with rank ≤ 10; the diagram for worldwide human population, for combinations with rank ≤ 20, is also shown in Figure 1b. Thus, the general picture of the contribution of epitopes was considered from the standpoint of the average_hit values.

### 3.2. Distribution of Conformational B-Cell Epitopes in the Tertiary Structure of IgA1 Protease

To justify the choice of the structure of the protective antigen, we examined the distribution of potential B-cell epitopes on the surface of the base molecule, using its 3D model as an input.

The 28–966 region, modeled with high accuracy according to pLDDT, has almost identical tertiary structure in 3D models of A^28^–P^1004^, MA^28^–P^1004^-LEH_6_ and ADC80147.1 (see Section 2.1.3)—this fact was defined by superimposing their Cα atom coordinates via Swiss PDB Viewer program (Figure S3): RMSD value for the A^28^–P^1004^ and MA^28^–P^1004^-LEH_6_ pair was 0.79 Å, for the ADC80147.1 and MA^28^–P^1004^-LEH_6_ pair—0.51 Å, and for the ADC80147.1 and A^28^–P^1004^ pair—0.99 Å, which shows the equivalence of these models for calculations related to tertiary structure of this area.

**Figure 2:**
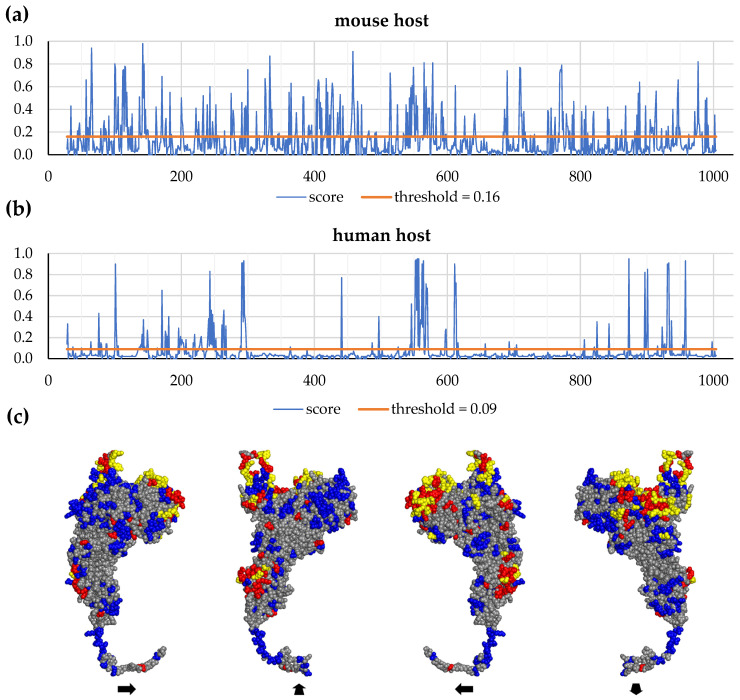
Distribution of amino acid residues included in the conformational B-cell epitopes, predicted in SEPPA 2.0 (for secreted antigen, with default thresholds) for the surface of the region A^28^–P^1004^ of IgA1 protease ADC80147.1, shown as 2D charts for mouse (**a**) and human (**b**) hosts, and selected on the model of the 3D structure (**c**). At the 3D model, amino acid residues that belong to the epitopes for both mouse and human hosts are colored yellow, other amino acid residues from epitopes for mouse host are colored blue, for human host—red, non-epitope residues—gray.

Figure 2 and Figure S4 show the data on the calculated distribution of the enzyme amino acid residues included in the predicted B-cell epitopes (see Section 2.1.4) for model of the tertiary structure of the IgA1 protease ADC80147.1 region A^28^–P^1004^. On this model we visually demonstrate the distribution of the conformational B-cell epitopes on the protein surface, as it was predicted in the SEPPA 2.0 program for mouse and human hosts (Figure 2c).

The data obtained by the ElliPro and DiscoTope 2.0 methods showed significant similarity (Figures S4 and S5).

The results of the SEPPA 2.0 program, taking into account the species of immune host and subcellular localization of protein antigen, for immunized mice showed an almost uniform distribution of amino acid residues included in the conformational B-cell epitopes. At the same time, for human hosts the amino acid residues that are parts of the conformational B-cell epitopes are localized mainly in N-, central and C-regions of A^28^–P^1004^.

### 3.3. Comparative Analysis of Tertiary Structures of Created Recombinant Proteins

In order for recombinant proteins created on the basis of selected fragments of IgA1 protease A^28^–P^1004^ to have immunogenic and protective activity close to the original enzyme, they must retain as many determinant conformational B-cell epitopes, located in the corresponding regions of the original enzyme, as possible. The issue that conformational B-cell epitopes, existing in the IgA1 protease A^28^–P^1004^, would be preserved in potential structures of recombinant proteins—is the issue we presume linked with the preservation of the 3D structure of the fragments, which make up these proteins, close to their 3D structure in the original enzyme. To determine the extent to which the spatial structures of the newly created compounds differ from the corresponding 3D structures in the protein 1, we carried out a comparative analysis of the models of tertiary structures of recombinant proteins 2–5 and of recombinant proteins we created earlier: ME^135^–H^328^-LEH_6_, MW^329^–H^622^-LEH_6_ and MH^835^–P^1004^-LEH_6_ with the model of the tertiary structure of protein 1.

**Figure 3:**
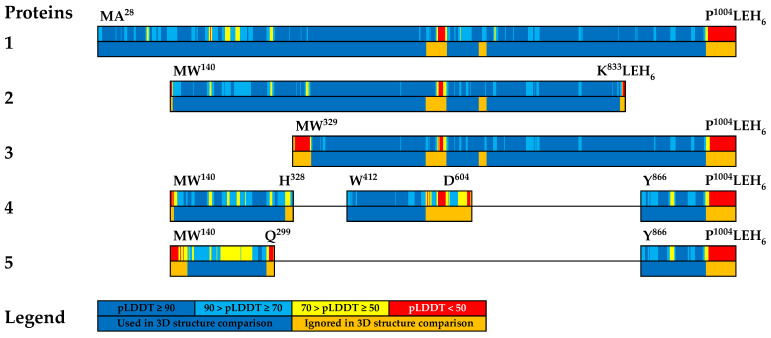
Two-level schematic representations of aligned proteins 1–5. The top halves of the schematic protein images are colored according to pLDDT values: blue for pLDDT ≥ 90 (high accuracy), light blue for 90 > pLDDT ≥ 70 (medium accuracy for side chains, good accuracy for backbone), yellow for 70 > pLDDT ≥ 50 (low accuracy), red for pLDDT < 50 (should not be interpreted: “is either unstructured in physiological conditions or only structured as part of a complex”, as stated AlphaFold FAQ at https://alphafold.ebi.ac.uk/faq, accession date 25 January 2024). The bottom halves are colored blue and orange, with the regions selected for RMSD-based matching being colored blue.

For this purpose, individual sections of the AlphaFold2 protein models were superimposed (by Cα atoms) using the Swiss PDB Viewer program, with the results presented as RMSD. The areas to superimpose were selected based on pLDDT (Figure 3 and Figure S6).

The overlay results are presented by RMSD in Table S1. It is obvious that, according to the calculated data, the sections of the models reliably predicted tertiary structures. We can conclude that for almost all considered regions that have presumably reliable models of tertiary structure, the RMSD values do not exceed 1.37 Å (Figure S6, Table S1). Thus, all presumably reliable regions of the truncated protein models we constructed retain the tertiary structure characteristic of the corresponding regions of the mature IgA1 protease. However, this does not apply to the model of the N-part of protein 5: according to pLDDT, this model is not reliable, and the results obtained using it (e.g., RMSD of 2.71 Å in Table S1) should be treated with caution.

### 3.4. Study of the Immunogenic and Protective Properties of the Created Recombinant Proteins

For carrying out biological tests, proteins 1–5 were obtained and purified. The length, molecular mass, pI (based upon amino acid pKa values from Bjellqvist et al. [41]) and preparation purity for these proteins was no less than 92% (see Table S2 and Figure S7).

**Figure 4:**
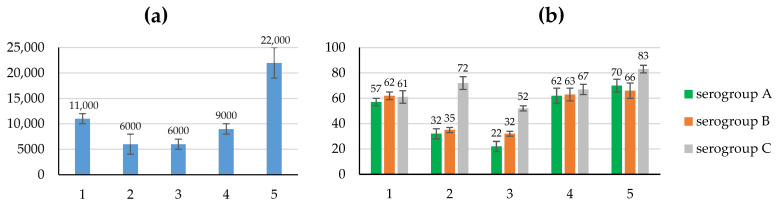
Immunogenic and protective properties of proteins 1–5. (**a**) Level of antibodies to protein 1, 1/titer; (**b**) degree of protection from *N. meningitidis* serogroups, %.

The immunogenic properties of recombinant proteins were assessed by the level of specific antibodies to protein 1 in the blood sera of immunized mice at the peak of antibody formation, and the protective properties and ability to form long-term immunological memory were assessed by the degree of protection of animals from infection with a live virulent culture of meningococci of serogroups A, B and C in later periods (4–6 months after immunization), in the absence of antibodies to protein 1 in the sera. Data on the level of specific antibodies in the blood sera of the BALB/c mice immunized with the obtained recombinant proteins, and the degree of protection (in %) of these animals after infection with meningococci, are given in Figure 4 and Table S3.

The level of antibodies to protein 1 in the sera of mice immunized with recombinant proteins 2 and 3, in the structure of which there is a common region W^329^–K^833^ from the central part of the protein 1 molecule, was similar and differed from that of the original protein by almost twofold (antibody titers, 10^3^/titer: 6 ± 2, 6 ± 1 and 11 ± 1, respectively, *p* < 0.001).

A decrease in titers of antibodies to truncated recombinant proteins 2 and 3 in the blood sera of immunized mice in comparison with those to the original protein 1 is consistent with a decrease in post-vaccination protection of the animals against infection with meningococci of serogroups A and B. Obviously, the level of protection of mice immunized with proteins 2 and 3 is significantly lower than that of the original protein 1: 32 ± 4%, 22 ± 4% and 57 ± 3%, respectively (*p* < 0.001), when infected with *N. meningitidis* serogroup A, and 35 ± 2%, 32 ± 2% and 62 ± 3%, respectively (*p* < 0.001), when infected with *N. meningitidis* serogroup B. At the same time, the protection of animals, immunized with these recombinant proteins, from infection with serogroup C meningococcus changes significantly less: 72 ± 5%, 52 ± 2% and 61 ± 5%, respectively (*p* < 0.01).

Despite the low level of antibodies to protein 1 in the blood serum of mice immunized with M(E^135^–H^328^)-LEH_6_ ((1.6 ± 0.3) × 10^3^/titer), the level of protection of immunized animals from infection with meningococci A, B and C was reliably higher than for immunization with the original protein 1: 72 ± 8%, 72 ± 7%, 77 ± 6% and 57 ± 3%, 62 ± 3%, 61 ± 5%, respectively (see ELISA and Protectivity for Table S3, *p* < 0.005). These conclusions were confirmed by studying the immunogenic and protective properties of hybrid recombinant proteins 4 and 5. Despite the significant differences in the primary structure of the recombinant protein 4 and the original protein 1, the levels of antibodies in the blood sera of animals immunized with these compounds remained virtually unchanged (antibody titers, 10^3^/titer: 9 ± 1 and 11 ± 1, respectively, *p* > 0.8). Moreover, for mice immunized with protein 4, the level of protection after infection with meningococci A, B and C (62 ± 6%, 63 ± 5% and 67 ± 4%, respectively) remained at the level of that for the original protein 1 (57 ± 3%, 62 ± 3% and 61 ± 5%, respectively, *p* > 0.8).

On the contrary, immunization of mice with recombinant protein 5, the primary structure of which includes only fragments of the N-terminal and C-terminal regions of protein 1, leads to an increase in titer of antibodies in the blood serum of animals immunized with protein 5, in comparison with protein 1 (antibody titer, 10^3^/titer: 22 ± 3 and 11 ± 1, respectively, *p* < 0.001).

In addition, in comparison with that for protein 1, the level of protection of mice immunized with protein 5 from infection with meningococci of serogroups A and serogroups C increased significantly (57 ± 3% and 70 ± 5%, 61 ± 5% and 83 ± 3%, respectively, *p* < 0.001).

## 4. Discussion

The recombinant IgA1 protease MA^28^–P^1004^-LEH_6_ has a high molecular mass (109,020.08 Da), a large number of potential antigenic determinants and, accordingly, a high antigenic load on the host body during immunization, which have led to the need to search for shorter immunogenic fragments of IgA1 protease A^28^–P^1004^, with an effective protective effect against meningococci of various serogroups. A candidate meningococcal vaccine should cause adaptive immune response, induce immunological memory [42], and protect the host from meningococcal infection. When selecting these potential fragments, we relied on previously obtained data on the immunogenic and protective properties of recombinant proteins containing fragments of IgA1 protease A^28^–P^1004^ from various parts of the molecule: ME^135^–H^328^-LEH_6_, MW^329^–H^622^-LEH_6_ and MH^835^–P^1004^-LEH_6_ [17], and the results of calculations on the distribution of B- and T-cell epitopes in the structure of this enzyme (Section 2.1.2 and Section 2.1.4).

The results of the of MHC-II T-cell epitope predictions for the BALB/c mice (see Section 3.1) correspond well with the protective antimeningococcal properties of recombinant ME^135^–H^328^-LEH_6_ and MH^835^–P^1004^-LEH_6_, but do not explain the lack of immunogenic and protective properties of MW^329^–H^622^-LEH_6_ [17]. The predicted distribution of MHC-II T-cell epitopes for human hosts is much more uniform than for BALB/c mouse hosts (see Figure 1), which limits the use of these data for selecting IgA1 protease A^28^–P^1004^ fragments promising for designing a structure of an active substance of the vaccine, like choosing these fragments more precisely, but not being too decisive about fragments as large as about a hundred residues.

The data obtained in Section 3.2 in the form of 2D charts are suitable for strategic selection of areas A^28^–P^1004^ as they indicate areas of high density of the amino acid residues that are highly capable of being parts of the conformational B-cell epitopes. They are localized mainly in the area the IgA1 protease fragments E^135^–H^328^, W^329^–H^622^ and H^835^–P^1004^ that are of interest to us, and they are practically absent in the interval K^623^–W^834^. The latter interval does not seem promising from this perspective.

Our previously obtained data [17] (see Introduction) on the study of the immunogenic and protective properties of the recombinant proteins created on the basis of fragments from various parts of the IgA1 protease A^28^–P^1004^ molecule made it possible to understand the importance of the presence of the N-terminal (E^135^–H^328^) and C-terminal (H^835^–P^1004^) regions of the enzyme in the structure of a potential protective antigen. We used truncated versions of these regions as parts of fusion proteins, in which they were covalently linked to each other either directly or through a linker from the truncated fragment W^329^–H^622^ from the central part of the IgA1 protease A^28^–P^1004^.

To study the role of the K^623^–K^833^ region of IgA1 protease A^28^–P^1004^ in inducing immunogenic and protective properties, we selected fragments of this enzyme with amino acid sequences W^140^–K^833^ and W^329^–P^1004^. The primary structure of W^329^–P^1004^ differed from the structure of the basic enzyme A^28^–P^1004^ by the absence of only the N-terminal region A^28^–H^328^, and the structure of W^140^–K^833^—by the absence of the N-terminal (A^28^–N^139^) and C-terminal (W^834^–P^1004^) regions of the original protease. Based on this, two recombinant proteins were proposed: protein 2 (with molecular mass 77,609.56 Da) and protein 3 (with molecular mass 74,819.44 Da).

While choosing the structure of fusion proteins, we took into account the fact that to ensure the solubility of potential recombinant proteins, the isoelectric point value should differ from the physiological pH value by at least 1 pH unit. For this purpose, the structure of the truncated sections included in the fusion proteins was selected by sequentially removing amino acid sequences, containing charged residues of glutamic and aspartic acids, arginine, lysine and histidine, from the C- and/or N-termini of fragments E^135^–H^328^, H^835^–P^1004^ and W^329^–H^622^, and further calculating the isoelectric point (pI) values for each variant of fusion protein. As a result, we selected several variants of fusion protein structures with the required pI values. To create a fusion protein from two truncated sections of the IgA1 protease A^28^–P^1004^, we chose the enzyme fragments W^140^–H^328^, W^412^–D^604^, and Y^866^–P^1004^ for protein 4 (molecular mass 59,407.63 Da), and W^140^–Q^299^ and Y^866^–P^1004^ for protein 5 (molecular mass 34,690.13 Da).

**Figure 5:**
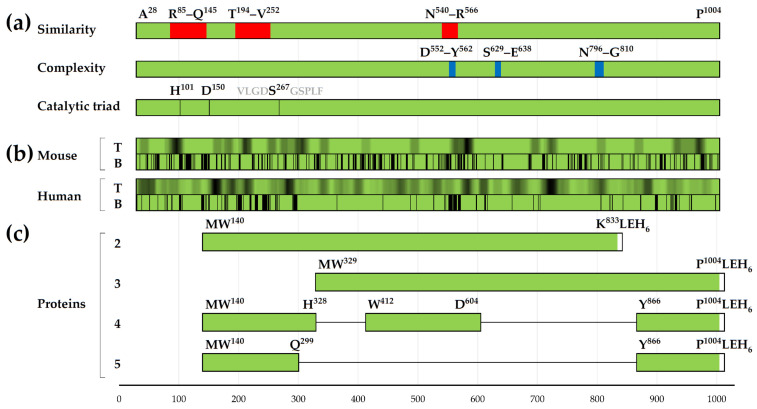
Simplified representation of recombinant proteins design based of sequence and epitope data for A^28^–P^1004^. (**a**) Sequence similarity (with IgA1 proteases from various strains of *N. meningitidis*) and low complexity regions data from [8]. Regions with low (see Table 2 of [8]) similarity are colored red. Low complexity regions are colored blue. In the catalytic triad of the serine IgA1 protease, Ser is shown as a part of VLGDSGSPLF sequence [7]. (**b**) T- and B-cell epitope prediction results for mouse and human hosts, based on Figure 1 and Figure 2. For T-cell epitopes, amino acid residues with corresponding epitope hit and average_hit for mouse and human hosts, respectively, are colored darker for higher values in a chart. For B-cell epitopes, amino acid residues with score above recommended threshold are colored black. (**c**) Schemes of recombinant proteins 2–5.

Data on the structures of the IgA1 protease A^28^–P^1004^ regions, selected for the creation of recombinant proteins with immunogenic and protective properties and using them as the main component of a potential meningococcal vaccine, were combined with data on the predicted distribution of amino acid residues included in the MHC-II T-cell epitopes (Figure 1) and conformational B-cell epitopes (Figure 2) for mouse and human hosts (based on the results of comparison of 2D charts from Figure S4, we used data from the SEPPA 2.0 program for human host). The summary of these data (with previously known information about similarity and complexity [8]) is presented here in shortened (Figure 5) and extended (Figure S5) forms.

Enzyme fragments with linear amino acid sequences W^140^–K^833^ and W^329^–P^1004^ contain all MHC-II T-cell epitopes and amino acid residues that potentially are part of the conformational B-cell epitopes in these intervals of IgA1 protease A^28^–P^1004^ (Figure 5 and Figure S5). It is obvious that the selection of the regions W^140^–Q^299^ and Y^866^–P^1004^ to create the fusion protein 5 and the regions W^140^–H^328^, W^412^–D^604^ and Y^866^–P^1004^ to create the fusion protein 4 leads to little loss of MHC-II T-cell epitopes and amino acid residues that potentially are part of the conformational B-cell epitopes of IgA1 protease A^28^–P^1004^, at least in N- and C-terminal regions of potential fusion proteins.

Previously, we showed [8] that the primary structures of the hypothetical mature IgA1 proteases from Gram-negative bacteria *N. meningitidis*, *N. gonorrhoeae* and about a quarter of the studied strains of *H. influenzae* (the latter also has other variants of IgA1 protease) have more than 85% similarity. Such a high degree of conservation of amino acid sequences is observed, in particular, in the intervals of the structure of IgA1 protease A^28^–P^1004^ that are of interest to us: 146–193, 253–539, 567–628 and 811–1004 (Figure 5a). It is obvious that the amino acid sequences W^140^–H^328^, W^412^–D^604^, and Y^866^–P^1004^, included in the primary structure of the recombinant fusion proteins 4 and 5, while also having non-conserved areas, largely fall into intervals with a high degree of identity of the primary structures of these proteins from the above-mentioned Gram-negative bacteria. These data suggest that the vaccine with the active component being recombinant proteins 4 and 5, as well as other proteins based on conserved fragments of IgA1 protease, will protect people from infection not only with meningococci, but also with other pathogens that produce serine IgA1 proteases with similar amino acid sequences.

Analysis of results of the study of immunogenic and protective properties of the created recombinant proteins 2 and 3 (see Section 3.4) on the BALB/c mouse model shows that the removal of either N-terminal, or both the N-terminal and the C-terminal regions from the primary structure of the original protein led to a noticeable decrease in antibody titer, thereby reducing the immunogenic and, for meningococci A and B, protective properties. This data, in combination with previously obtained data on the properties of recombinant proteins ME^135^–H^328^-LEH_6_, MW^329^–H^622^-LEH_6_ and MH^835^–P^1004^-LEH_6_ [15,17], confirm the idea that the structure of an antimeningococcal vaccine preparation, based on fragments of the primary structure of protein 1, should include fragments of both the N-terminal and C-terminal regions of the original protein 1. On the contrary, the W^329^–K^833^ fragment from the central part of the primary structure of protein 1, including the amino acid sequence of the previously unexplored region K^623^–W^834^, presented in a vaccine preparation did not have a positive effect on either the immunogenic or protective properties of the recombinant proteins.

At the same time, a key role in animal protection is most likely played by the protein 1 molecule N-terminal part fragment E^135^–H^328^, which is a part of the recombinant protein M(E^135^–H^328^)-LEH_6_ (molecular mass 23,366.74 Da) [17]. But it contains sequence regions that differ in *N. meningitidis* IgA1 proteases of type 1 (which IgA1 protease ADC80147.1 belongs to) and type 2. In the context of designing a universal vaccine against meningococci producing IgA1 protease type 1 or type 2, this requires us to include in the hybrid molecule an additional region that has significant immunogenic and protective properties and is highly conservative regardless of the type of IgA1 protease, serogroup, etc. The immunogenic and protective properties of proteins 4 and 5 on the BALB/c mouse model are characterized in Figure 4 and Table S3. Immunogenic and protective properties of protein 4 are very close to those of protein 1. Protein 5 has higher immunogenic properties, and, for meningococci A and C, protective properties, and relatively similar protective properties for meningococci B. This may indicate that the structure of recombinant proteins 4 and 5 retain almost all the necessary MHC-II T-cell epitopes and conformational B-cell epitopes of protein 1. At the same time, the structural features of the recombinant protein 5 did not in any way affect the level of protection of mice immunized with it from infection with meningococci serogroup B.

The results of the study of the immunogenic and protective properties of the recombinant proteins we obtained allowed us to consider hybrid proteins 4 and 5 as the basis of a candidate vaccine against meningococcal infection.

We see the next step in this research as the development of conditions for obtaining liposomal preparations containing recombinant proteins 4 or 5, as well as DNA or RNA molecules encoding them in eukaryotic cells.

## 5. Conclusions

To justify the selection of fragments of the mature IgA1 protease A^28^–P^1004^ from *Neisseria meningitidis* serogroup B strain H44/76 and, on their basis, to the design and construct antigens for an antimeningococcal vaccine, with immunogenic properties and a protective antimeningococcal effect, we carried out calculations to predict the location of B- and T-cell epitopes in the enzyme structure.

Using the calculation data and taking into account the biological properties of the previously obtained recombinant proteins ME^135^–H^328^-LEH_6_, MW^329^–H^622^-LEH_6_ and MH^835^–P^1004^-LEH_6_, we selected the enzyme regions that could become potential vaccine antigens. On the basis of these regions, we constructed linear and hybrid structures with the necessary physicochemical properties, and we used these structures to create recombinant proteins. In experiments on the BALB/c mice, we studied the immunogenic and protective properties of the obtained recombinant proteins. Based on the test results, we selected proteins 4 (M(W^140^–H^328^)-(W^412^–D^604^)-(Y^866^–P^1004^)-LEH_6_) and 5 (M(W^140^–Q^299^)-(Y^866^–P^1004^)-LEH_6_) that were candidates for the role of the active ingredient of a universal monovaccine. These proteins showed a significant number of predicted epitopes in both humans and mice, therefore evaluation of biological activity in the BALB/c mice suggests relevance to humans.

The obtained results indicate the possibility of creating proteins that have high immunogenic and protective properties and are based on fragments of IgA1 protease A^28^–P^1004^; the high degree of conservativity of the used fragments of IgA1 proteases from *N. meningitidis*, *Neisseria gonorrhoeae* and a number of strains of *Haemophilus influenzae* allows us to suggest that such a vaccine can effectively protect humans from infection with these pathogens.

## 6. Patents

Patent RU 2701964 C2 “Recombinant protein having protective action on meningococcus (embodiments), polynucleotide coding recombinant protein, recombinant plasmid DNA containing said polynucleotide, host cell containing said recombinant plasmid DNA, method of producing recombinant protein” for proteins M(W^140^–H^328^)-(W^412^–D^604^)-(Y^866^–P^1004^)-LEH_6_ and M(W^140^–Q^299^)-(Y^866^–P^1004^)-LEH_6_ was registered on 2 October 2019.

## Data Availability

The original data presented in the study are either openly available in the Supplementary Materials (source data for the diagrams, extended comparison of data), or are available upon reasonable request from the corresponding author (if more details are required).

## Supplementary Materials

The following supporting information can be downloaded at: https://www.mdpi.com/article/10.3390/vaccines12121355/s1, Figure S1: The rank values for the predicted combinations of MHC-II T-cell epitopes, located on the A^28^–P^1004^ chain, with alleles of BALB/c mouse; rank ≤ 20; Figure S2: (a) The average_hit value sums for amino acid residues involved in the predicted combinations of MHC-II T-cell epitopes, located on the A^28^–P^1004^ chain, with alleles of human worldwide populations; rank ≤ 20; (b) The average_hit value sums for amino acid residues involved in the predicted combinations of MHC-II T-cell epitopes, located on the A^28^–P^1004^ chain, with alleles of human worldwide populations; rank ≤ 10; Figure S3: Region 28–966 overlay of IgA1 protease unrelaxed 3D models built in AlphaFold2; Figure S4: 2D charts of the distribution of amino acid residues included in the predicted conformational B-cell epitopes on the surface of the region A^28^–P^1004^ of IgA1 protease, with default thresholds; Figure S5: Summary for truncated protein structure and predicted T- and B-cell epitopes; Figure S6: Two-level schematic representations of the studied proteins; Figure S7: Gel image of the results of SDS-PAGE of proteins MA^28^–P^1004^-LEH_6_ (protein 1), MW^140^–K^833^-LEH_6_ (protein 2), MW^329^–P^1004^-LEH_6_ (protein 3), M(W^140^–H^328^)-(W^412^–D^604^)-(Y^866^–P^1004^)-LEH_6_ (protein 4) and M(W^140^–Q^299^)-(Y^866^–P^1004^)-LEH_6_ (protein 5); Table S1: RMSD (by Cα atoms) between the selected sections of the relaxed models of the tertiary structure of the studied recombinant proteins; Table S2: Properties of IgA1 protease MA^28^–P^1004^-LEH_6_ and recombinant proteins created based on its fragments; Table S3. Immunogenic and protective properties of the obtained recombinant proteins.
